# Strangulated Small Bowel Internal Hernia Under the External Iliac Artery After Robotic Cystoprostatectomy

**DOI:** 10.7759/cureus.39837

**Published:** 2023-06-01

**Authors:** Aizaz Khalid, Mohamed A Salman, Simon Woodhams, Richard C Newton

**Affiliations:** 1 General Surgery, University Hospitals Sussex NHS Foundation Trust, Chichester, GBR; 2 Urology, University Hospitals Sussex NHS Foundation Trust, Chichester, GBR

**Keywords:** iliac artery, pelvic lymph node dissection, strangulated internal hernia, acute abdomen, cystoprostatectomy

## Abstract

Small bowel obstruction is a common surgical pathology encountered in the emergency department. The most common cause of small bowel obstruction is adhesions secondary to previous abdominal surgery. While strangulated external hernias are also a common cause of obstructions encountered, internal hernias leading to obstruction are rare. We present a 76-year-old male who presented with an acute abdomen and was later diagnosed with an internal hernia underneath his right external iliac artery.

Internal herniation underneath the iliac vasculature is a recent phenomenon encountered very rarely after the natural anatomy has been disturbed in patients who have undergone pelvic lymph node dissection. Patients with a previous history of pelvic lymph node dissection should be suspected of having an internal hernia if they present with an acute abdomen. Closure of the peritoneum should also be considered in these patients as it may help prevent herniation.

## Introduction

Internal hernias were previously considered a rare cause of small bowel obstruction, but their incidence has been increasing in the past two decades [1]. This is partly due to increased detection but likely also due to increased use of laparoscopic and robotic surgery for abdominopelvic procedures which makes closure of internal defects more challenging. Although the overall incidence is less than 1%, they account for up to 5.8% of all cases of small bowel obstruction. Ghahremani devised a classification for internal hernias which includes six groups: paraduodenal hernias (most common), hernias through the foramen of Winslow, transmesenteric hernias, pericecal hernias, intersigmoid hernias and perivesical hernias [2]. We present a 76-year-old who presented with a rare internal herniation of the small bowel underneath the iliac vasculature, which does not fall under any of these six groups. 

## Case presentation

A 76-year-old gentleman presented to the emergency department with rapid-onset severe constant abdominal pain after eating. It migrated from the umbilical region to the right iliac fossa (RIF). He had opened his bowels in the morning and did not complain of any vomiting, nausea, or fevers. Whilst very active and lean, he took apixaban for atrial fibrillation and, three years previously, had undergone neoadjuvant chemotherapy and robot-assisted cystoprostatectomy with the formation of an ileal conduit urostomy for an invasive bladder transitional cell carcinoma with an incidental prostate carcinoma. He is a non-smoker with no history of chronic cough or other risk factors for herniation. 

**Figure 1:**
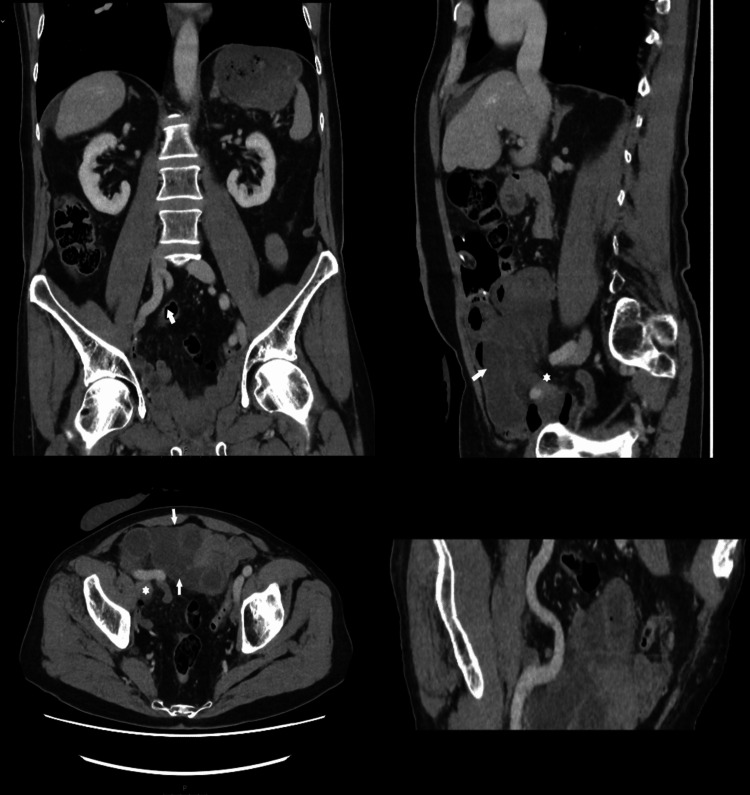
Computerized tomography (CT) images show that the right external iliac artery takes an abnormal medial turn after bifurcation of the right common iliac. This is denoted by the arrow in the top-left coronal film and more accurately in the reconstructed angulated film at the bottom-right. Axial film (bottom-left) shows strangulated bowel loops (arrows) herniating underneath the abnormal right external iliac artery. Loops proximal to the herniation point can also be seen here (asterisk). The sagittal film in the top-right shows the herniation point (asterisk) and the herniated bowel loops (arrow).

He had tenderness in the umbilical region and RIF with rebound tenderness. The urostomy was working. Though his pulse and inflammatory markers were within normal range, his serum lactate was 5.6mmol/L. A contrast-enhanced CT scan of the abdomen and pelvis showed dilated small bowel loops, a portion of which were poorly enhanced, suggesting a closed loop small bowel obstruction (Figure 1). The radiologist also reported a prominent iliac artery. It was assumed that the obstruction would be from an adhesive band in relation to the ileal conduit, a mesenteric window formed during the creation of the conduit, or if very unlucky, a ureter.

**Figure 2:**
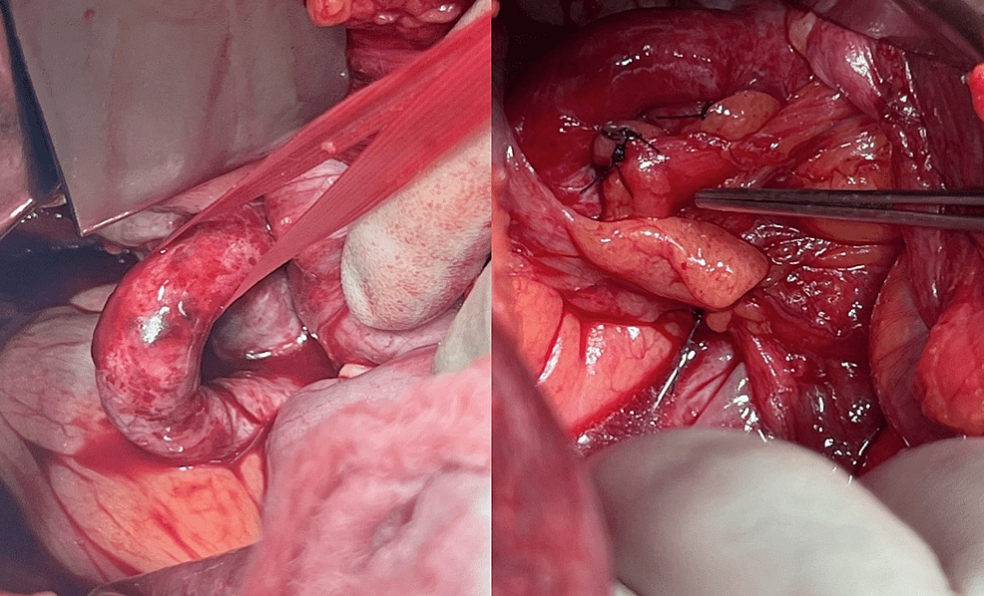
Tortous right external iliac artery with a defect (left) underneath through which small bowel was found herniated. The defect was closed with appendices epiploicae and peritoneum as shown in the figure on the right.

He was resuscitated with intravenous fluids and broad-spectrum antibiotics and had a diagnostic laparoscopy. The findings were necrotic small bowel and haemorrhagic fluid but no obvious cause was found. A decision was made to convert to a lower midline laparotomy. The ileal conduit and ureters were found to be intact and patent. Approximately 40 cm of ileum herniated from lateral to medial between the pelvic brim and a very tight, thick cord-like band. The bowel was necrotic and contained a large amount of bloody fluid. Only after a very awkward reduction of the necrotic bowel did the “band” become appreciably pulsatile; it was a looping right skeletonized external iliac artery. The necrotic small bowel was resected, and bowel continuity was established with a stapled side-to-side anastomosis. The hernia defect (Figure 2) was closed by attaching a combination of sigmoid colon appendices epiploicae and mobilized pelvic sidewall peritoneum to the arterial adventitia with a 3/0 PDS suture. The patient made an excellent recovery, spending two days in critical care before being discharged on Day 8.

## Discussion

Internal herniation of small bowel loops underneath the iliac arteries is a rare phenomenon that has been reported in a few previous case reports [3-14]. The first instance of internal herniation was reported by Guba et al. [3] in 1978. Since then, it has been reported at least 11 more times, and it seems to be occurring more frequently as 10 of the 12 reports we examined are from the last 10 years. Our patient had undergone a robotic cystoprostatectomy with pelvic lymph node dissection, similar to all the case reports we examined. All patients who have internal herniation under the iliac plexus had a past surgical history of pelvic lymph node dissection. 

Pelvic lymph node dissection is the most effective method of detecting lymph node metastasis in several pelvic malignancies [15]. Harvesting lymphatic tissue close to the iliac vasculature leads to a significant space that can form the basis of internal herniation. In our experience, we found that the external iliac was unusually tortuous and took a medial turn which was possibly due to prolonged pressure effects on the vessel by the bowel occupying this space. Currently, there are no guidelines in place on how to deal with this free space after lymph node dissection. Closure of the peritoneum during index surgery could be considered as a preventive measure against internal herniation. Some studies have looked at the benefits of closure vs nonclosure of the peritoneum in pelvic surgeries but have been inconclusive [16]. 

After the reduction of the herniating content, we found a large hernial defect underneath the external iliac artery (Figure 2). Addressing this defect presented a major challenge in the absence of established guidelines on how to deal with such a defect. We used a combination of peritoneum and colonic appendices to close the defect. Pridjian et al. [7] also used peritoneal flaps to close the defect. In our review of the literature, we found that various methods have been employed in this regard, ranging from leaving the defect open [5,10-11,14] to peritoneal grafting [3,4], continuous suturing [12,13], use of collagen patch [6] and fixation of the artery to the lateral peritoneal wall [8].

## Conclusions

Internal herniation underneath the iliac vasculature in a rare phenomenon that occurs after skeletonization of these vessels due to pelvic lymph node dissection. This diagnosis should be considered in patients presenting with symptoms of bowel obstruction following pelvic surgery, especially where there are tortuous iliac vessels. Closure of peritoneum could be considered to potentially avoid its complication. More studies are needed to understand the true incidence of internal hernias after pelvic lymph node dissection.
